# The clinical impact of mitochondrial autophagy on very late-onset recurrence after catheter ablation for atrial fibrillation

**DOI:** 10.1093/ehjopen/oeaf058

**Published:** 2025-05-17

**Authors:** Keisuke Uchida, Naoya Kataoka, Teruhiko Imamura, Takahisa Koi, Koichiro Kinugawa

**Affiliations:** Second Department of Internal Medicine, University of Toyama, 2630 Sugitani, Toyama 930-0194, Japan; Second Department of Internal Medicine, University of Toyama, 2630 Sugitani, Toyama 930-0194, Japan; Second Department of Internal Medicine, University of Toyama, 2630 Sugitani, Toyama 930-0194, Japan; Second Department of Internal Medicine, University of Toyama, 2630 Sugitani, Toyama 930-0194, Japan; Second Department of Internal Medicine, University of Toyama, 2630 Sugitani, Toyama 930-0194, Japan

**Keywords:** Atrial fibrillation, Catheter ablation, Mitochondria, Autophagy

## Abstract

**Aims:**

The mechanisms underlying very late-onset atrial fibrillation (AF) recurrence, defined as occurring more than 1 year after catheter ablation, are hypothesized to differ from those responsible for recurrence within the first year; however, this remains uncertain.

**Methods and results:**

Two investigations were conducted in patients undergoing AF ablation. First, non-targeted metabolome analysis was performed in 10 patients with very late-onset recurrence and 10 without recurrence. Second, based on metabolomic findings implicating autophagy, serum levels of the autophagy-related proteins Parkin, a marker of mitophagy, and ATG5, an indicator of bulk autophagy, were measured using ELISA. Associations between these variables and very late-onset recurrence were analysed. Among the 203 patients (mean age 70 years, 63% male), 16 experienced very late-onset recurrence during a mean follow-up of 954 days. Metabolome analysis identified 255 peaks (177 cations and 78 anions). Principal component analysis revealed a reduction in γ-glutamyl dipeptides, contributors to mitochondrial autophagy, in the recurrence group. A serum Parkin level below the median was independently associated with very late-onset recurrence (hazard ratio 3.82, 95% confidence interval 1.20–12.13, *P* = 0.023), after adjustment for left atrial diameter and diabetes mellitus. In contrast, ATG5 levels were not significantly associated. Parkin levels did not predict recurrence within the first year (log-rank *P* = 0.09).

**Conclusion:**

Reduced serum Parkin levels were independently associated with very late-onset recurrence following AF ablation, suggesting that impaired mitochondrial autophagy may contribute to the pathogenesis of long-term AF recurrence.

## Introduction

The number of patients with atrial fibrillation (AF) has been increasing worldwide each year, leading to a higher risk of thromboembolism and mortality.^1^ Catheter ablation is a widely accepted curative therapeutic strategy for AF; however, it is well established that approximately half of patients experience recurrence during long-term follow-up after ablation.^2^ Therefore, patients at high risk of stroke, as indicated by elevated CHA2DS2-VASc scores, are recommended to continue anticoagulant therapy even after successful catheter ablation.^3^ However, there are cases of bleeding associated with the continuation of anticoagulant therapy, and it is concluded that no treatment surpasses the maintenance of sinus rhythm in patients with AF.^4^

The causes of AF recurrence following catheter ablation are attributed to several factors, which vary depending on the timing of recurrence after the procedure. It is well established that the reconnection of pulmonary veins is the primary cause of AF recurrence within 1 year following ablation; however, very late-onset recurrence after the procedure is often associated with non-pulmonary vein foci.^5^ The CHA2DS2-VASc score, which has been reported to be associated with left atrial remodelling at higher values, is recognized as a predictive marker for late recurrence, defined as recurrence occurring more than 1 year after the procedure.^6,7^ These findings suggest that the atria become progressively more susceptible to the onset and maintenance of AF as a consequence of remodelling. Therefore, it is essential to identify the factors contributing to atrial remodelling, such as oxidative stress, as this may be helpful for risk stratification.^8^

Recent literature has demonstrated the utility of non-targeted metabolomics methods in identifying key factors involved in the pathophysiology associated with coronary artery disease and heart failure; however, evidence regarding its application in AF recurrence remains limited.^9^

Therefore, in this study, we proposed a research design consisting of the following two components: (i) For the feasibility study, we conducted a metabolomic analysis in patients with AF recurrence more than 1 year after ablation and in those without recurrence, assessing metabolites associated with recurrence. (ii) Subsequently, we measured the blood concentrations of proteins involved in the metabolic pathways identified through metabolomic analysis and investigated whether these proteins could serve as predictors of late-onset AF recurrence across numerous ablation cases.

## Methods

### Study population

The present study was conducted in compliance with the Declaration of Helsinki and was approved by the institutional review board at the University of Toyama. Written informed consents were obtained from all subjects. Patients who were hospitalized for AF ablation at our institution between September 2019 and January 2023 were prospectively screened. The diagnosis of AF was based on the presence of AF waveforms on 12-lead surface electrocardiograms recorded within 1 year prior to screening.

The inclusion criteria were as follows: (i) age 20 years or older, (ii) patients who underwent AF ablation, and (iii) subjects who consented to participate. Exclusion criteria included the following: (i) age under 20 years, (ii) patients who underwent cardiac intervention within the past 3 months, (iii) patients with end-stage renal disease requiring dialysis, (iv) patients unable to administer anticoagulants, and (v) patients lost to follow-up within 3 months, which corresponds to the blanking period following the procedure.

For the non-targeted metabolomic analysis, which was the first objective of the study, consecutive cases were selected until 10 cases were identified in each group: those with recurrence more than 1 year after ablation and those without recurrence during the observation period (median: 1115 days; interquartile range: 848–1458 days). All enrolled cases were then used for the analysis aimed at identifying predictors of AF recurrence, which constituted the second objective of the study.

### Data and samples collected at the time of enrolment

Clinical characteristics at baseline, including demographic information, electrocardiographic and echocardiographic parameters, and laboratory data at the time of admission, were obtained from the electronic medical record. Blood samples were collected by plain blood tube on the day prior to the procedure. Serum samples were separated by centrifugation at 2000 × *g* for 20 min. Separated serum samples were stored frozen at −80°C in silicone-coated tubes until analysis.

### Metabolomic analysis

The samples were analysed by Human Metabolome Technologies Inc. (HMT; Tsuruoka, Yamagata, Japan). Metabolite analysis was conducted using capillary electrophoresis time-of-flight mass spectrometry in accordance with HMT’s Scan Package. Data analysis included Welch’s *t*-test and Fisher’s exact test to assess significant differences between the two groups. Principal component analysis was performed to classify similar factors based on the distance between peaks, using the unweighted pair group method with arithmetic mean.

### Measurement of autophagy-related protein levels

Based on the findings of the metabolomic analysis, we directed our investigation towards mitochondrial autophagy, commonly referred to as mitophagy. Serum levels of Human Parkinson Disease Protein 2 (Parkin), a representative marker of mitophagy, and Human Autophagy Protein 5 (ATG5), a representative marker of bulk autophagy, were measured in all enrolled subjects using two commercially available ELISA kits (MBS732278 and MBS7209535, respectively; My BioSource, San Diego, CA, USA).^10,11^

### Ablation procedures

Catheter ablation was performed using either the EnSite system (Abbott Inc., Chicago, IL, USA) or the CARTO system (Biosense Webster Inc., Irvine, CA, USA) under sedation with continuous infusion of propofol and dexmedetomidine. The choice of ablation energy source, either radiofrequency or cryoballoon (Arctic Front Advance™, Medtronic Inc., Minneapolis, MN), was made at the operator’s discretion.

Continuous heparin infusion was administered to maintain an activated clotting time of over 300 s. In cases of radiofrequency ablation, pulmonary vein isolation was performed with a power setting of 50 W, either for 15 s using the EnSite system or until an Ablation Index of 500 was achieved using the CARTO system, except in regions where the oesophageal temperature exceeded 40°C. In cases of cryoballoon ablation, the procedure was performed for 120 s after the time required for isolation, or for the maximum of 240 s.

Additional linear ablation, such as roof line, mitral isthmus block line, or left atrial posterior wall isolation, was performed at the discretion of the operators. Linear ablation using radiofrequency energy was carried out with the settings described above. Extra-pulmonary vein ablation using a cryoballoon was limited to the left atrial roof and was achieved by delivering cryo-energy for 180 s at each of three contiguous sites. Cavotricuspid isthmus block line was created using radiofrequency ablation in cases where it was confirmed by the electrocardiogram before the procedure or induced during the procedure. Provocation testing for non-pulmonary vein triggers using isoproterenol was not performed.

### Clinical outcomes

The primary endpoint was AF recurrence, defined as the incidence of atrial tachyarrhythmias including AF, atrial flutter, and atrial tachycardia, occurring more than 1 year after the procedure, classified as the very late-onset recurrence. Atrial tachyarrhythmias were identified through a 12-lead surface electrocardiogram, 24 h or 2-week Holter monitoring, or a portable electrocardiograph (OMRON Corp., Kyoto, Japan). The secondary endpoint was defined as the incidence of atrial tachyarrhythmias occurring beyond 90 days after the procedure, classified as any recurrence.

### Statistical analysis

The data are presented as median with interquartile range for variables. Categorical variables are expressed as counts and percentages. Log transformation was applied to the analysis of biomarkers that exhibited non-normal distributions. Kaplan–Meier survival curves were generated, and the log-rank test was used to assess differences between groups. Cox proportional hazards regression analysis was performed to identify factors associated with the primary endpoint. The multivariable analysis was adjusted for variables with a *P* < 0.05 in the univariable analysis. Statistical analysis was performed using JMP version 14 (SAS, NC, USA).

## Results

### Baseline characteristics

A total of 203 subjects were enrolled in the study (*Table 1*). The median age was 70 years, and 75 subjects (37%) were female. Among the participants, 84 (41%) were classified as having persistent AF. A total of 65 subjects (32%) had a history of hospitalization for heart failure, with a median left ventricular ejection fraction of 63%. The left atrial diameter was mildly enlarged, with a median value of 42 mm among the enrolled subjects.

**Table 1:** Baseline characteristics

Variable	Overall (*n* = 203)
Demographics	
Age (years)	70 [61–76]
Female, *n* (%)	75 (37)
Body mass index (kg/m^2^)	23.4 [20.8–26.0]
Persistent atrial fibrillation, *n* (%)	84 (41)
CHA2DS2-VASc	3 [1–4]
Lifestyle	
Daily drinking	55 (27)
Daily smoking	32 (16)
Comorbidities	
History of heart failure admission, *n* (%)	65 (32)
Hypertension, *n* (%)	113 (56)
Diabetes mellitus, *n* (%)	31 (15)
Ischaemic stroke, *n* (%)	18 (9)
Vascular disease, *n* (%)	4 (2)
Aetiology of heart disease	
Ischaemia, *n* (%)	14 (7)
Dilated cardiomyopathy, *n* (%)	23 (11)
Valvular heart disease, *n* (%)	15 (7)
Cardiac implantable electronic devices, *n* (%)	6 (3)
Pacemaker, *n* (%)	5 (2)
Cardiac resynchronization therapy, *n* (%)	1 (0)
Medications	
ACEi or ARB, *n* (%)	96 (47)
ARNI, *n* (%)	10 (5)
β-Blockers, *n* (%)	130 (64)
Mineralocorticoid receptor antagonists, *n* (%)	35 (17)
Sodium-glucose cotransporter-2 inhibitors, *n* (%)	26 (13)
Echocardiographic parameters	
Left atrial diameter (mm)	42 [36–46]
LV ejection fraction (%)	63 [54–69]
Laboratory data	
Serum creatinine (mg/dL)	0.87 [0.72–1.00]
N-terminal pro-brain natriuretic peptide (pg/mL)	438 [135–1002]

The data are presented as median with interquartile range for variables. Categorical variables are expressed as counts and percentages.

ACE, angiotensin converting enzyme inhibitor; ARB, angiotensin receptor blocker; ARNI, angiotensin receptor neprilysin inhibitor; LV, left ventricular.

### Ablation procedures and outcomes

Radiofrequency ablation was performed in 84 subjects (41%), while the remaining subjects underwent cryoballoon ablation (*Table 2*). Pulmonary vein isolation was successfully achieved in all enrolled subjects, regardless of the energy source. Pulmonary vein isolation alone was performed in 137 subjects (67%), additional posterior wall isolation in 35 subjects (17%), and roof line isolation with cryoballoon in 31 subjects (15%). Anti-arrhythmic drugs were administered post-procedure in 30 subjects (15%), at the discretion of the attending physician.

**Table 2:** Therapeutic details

Variable	
Sources of ablation energy	
Radiofrequency, *n* (%)	84 (41)
Cryoballoon, *n* (%)	119 (59)
Strategies of the procedures	
Pulmonary veins isolation only, *n* (%)	137 (67)
Additional lines	
Posterior wall isolation, *n* (%)	35 (17)
Roof block line utilized by cryoballoon, *n* (%)	31 (15)
Cavotricuspid isthmus block line, *n* (%)	19 (9)
Superior vena cava isolation, *n* (%)	5 (2)
Medications at discharge	
Any anti-arrhythmic drugs, *n* (%)	30 (15)
Sodium channel blockers, *n* (%)	7 (3)
Bepridil, *n* (%)	7 (3)
Amiodarone, *n* (%)	16 (8)

The data are presented as median with interquartile range for variables. Categorical variables are expressed as counts and percentages.

**Figure 1:**
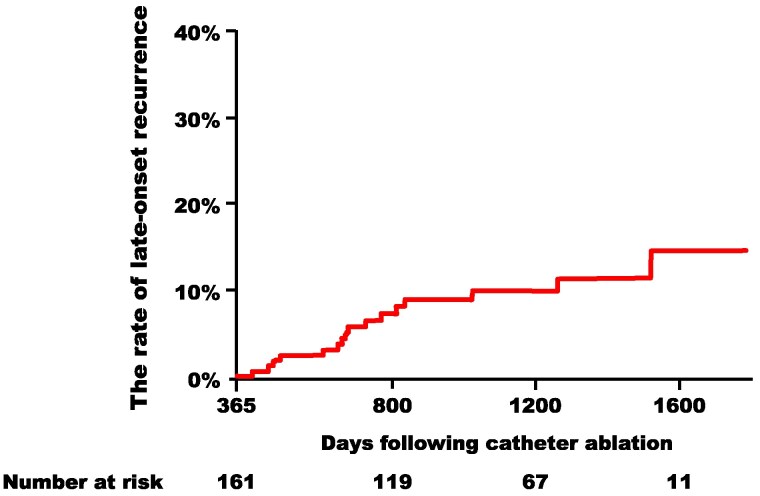
Kaplan–Meier curves for very late-onset recurrence in all enrolled subjects.

During a median follow-up of 954 days (range: 649–1325 days), the very late-onset recurrence occurred in 16 subjects (9.9%) (*Figure 1*), with no significant difference observed between radiofrequency-based or cryoballoon-based ablation (log-rank *P* = 0.54). The first 10 consecutive cases in this group were provided as samples for metabolomic analysis. The secondary endpoint was observed in 57 subjects (28%), again with no significant difference between the two ablation modalities (log-rank *P* = 0.96).

### The results of the metabolomic analysis

The baseline characteristics of the 20 subjects included in the metabolomic analysis (median age: 72 years, 12 males) were comparable between the very late-onset recurrence and recurrence-free groups (*Table 3*). The comparisons are as follows: median age (73 vs. 68 years, *P* = 0.23), male sex [8 (80%) vs. 4 (40%), *P* = 0.07], persistent AF type [8 (80%) vs. 4 (40%), *P* = 0.07], and median left atrial diameter [47 (42–50) mm vs. 43 (33–49) mm, *P* = 0.27], respectively.

**Table 3:** Comparison of baseline characteristics for the metabolomic analysis

Variable	The very late-onset recurrence	Recurrence-free	*P*-value
Demographics			
Age (years)	73 [69–80]	68 [54–80]	0.226
Female, *n* (%)	2 (20)	6 (60)	0.068
Body mass index (kg/m^2^)	21.8 ± 3.1	22.9 ± 3.3	0.545
Persistent atrial fibrillation, *n* (%)	8 (80)	4 (40)	0.068
CHA2DS2-VASc	3 [2–5]	2 [1–4]	0.203
Lifestyle			
Daily drinking	7 (70)	7 (70)	1.000
Daily smoking	1 (10)	2 (20)	0.531
Comorbidities			
History of heart failure admission, *n* (%)	3 (30)	3 (30)	1.000
Hypertension, *n* (%)	8 (80)	5 (50)	0.160
Diabetes mellitus, *n* (%)	3 (30)	1 (10)	0.264
Ischaemic stroke, *n* (%)	2 (20)	0 (0)	0.136
Vascular disease, *n* (%)	0 (0)	0 (0)	Not applicable
Aetiology of heart disease			
Ischaemia, *n* (%)	0 (0)	0 (0)	Not applicable
Dilated cardiomyopathy, *n* (%)	1 (10)	2 (20)	0.531
Valvular heart disease, *n* (%)	1 (10)	1 (10)	1.000
Cardiac implantable electronic devices, *n* (%)	0 (0)	0 (0)	Not applicable
Pacemaker, *n* (%)	0 (0)	0 (0)	Not applicable
Cardiac resynchronization therapy, *n* (%)	0 (0)	0 (0)	Not applicable
Medications			
ACEi or ARB, *n* (%)	6 (60)	4 (40)	0.371
ARNI, *n* (%)	0 (0)	0 (0)	Not applicable
β-Blockers, *n* (%)	4 (40)	8 (80)	0.068
Mineralocorticoid receptor antagonists, *n* (%)	1 (10)	3 (30)	0.264
Sodium-glucose cotransporter-2 inhibitors, *n* (%)	4 (40)	3 (30)	0.639
Echocardiographic parameters			
Left atrial diameter (mm)	47 [42–50]	43 [33–49]	0.256
LV ejection fraction (%)	61 [58–70]	57 [43–69]	0.150
Laboratory data			
Serum creatinine (mg/dL)	0.91 [0.71–1.03]	0.85 [0.70–1.05]	0.650
N-terminal pro-brain natriuretic peptide (pg/mL)	594 [253–914]	914 [168–2895]	0.450

The abbreviations are the same as those in *Table 1*.

**Figure 2:**
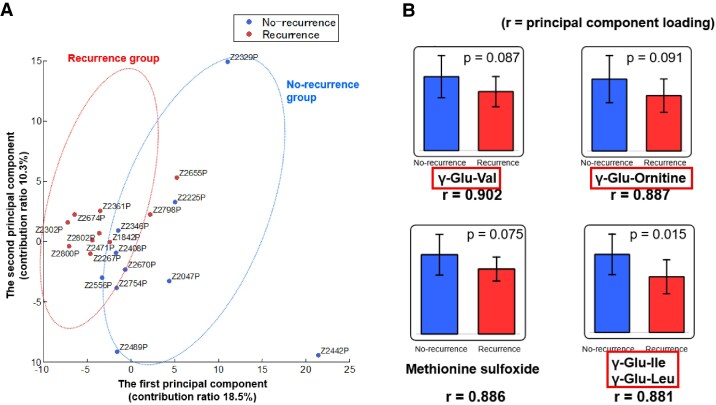
The results of metabolome analysis. (*A*) Principal component analysis. (*B*) The top four metabolites in the principal component loadings. Recurrence: very late-onset recurrence after ablation; No-recurrence: none.

Metabolomics analysis identified candidate compounds corresponding to 255 peaks (177 cations and 78 anions). The results of the principal component analysis demonstrated that the first principal component accounted for 18.5% of the variance (*Figure 2A*). Among the top four metabolites in the principal component loadings, three were classified as γ-glutamyl dipeptides, while the remaining metabolite was methionine sulfoxide, which is associated with oxidative stress (*Figure 2B*). The γ-glutamyl dipeptides showed a trend towards suppression in the very late-onset recurrence group compared to the recurrence-free group. Glutamine metabolism has been identified as a key contributor to mitophagy; inhibition of glutamine metabolism suppresses the activation of BNIP3, a well-established marker of mitophagy.^12,13^ Therefore, we aimed to investigate the predictive value of autophagy-related proteins in relation to very late-onset recurrence following AF ablation, focusing specifically on Parkin as a representative marker of mitophagy and ATG5 as a marker of bulk autophagy.

### Autophagy-related biomarkers and the primary endpoint

**Figure 3:**
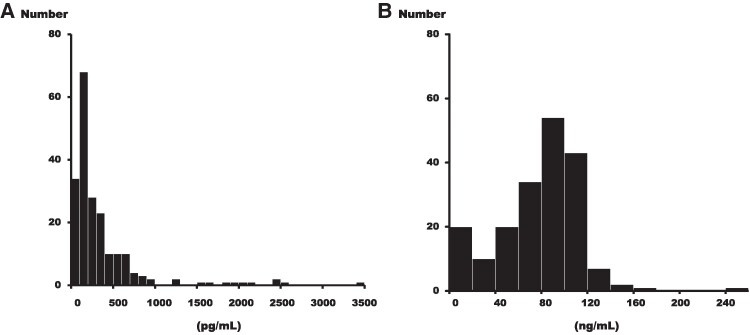
The distribution of Parkin and ATG5 levels. (*A*) Plasma level of Parkin. (*B*) Plasma level of ATG5. ATG5, autophagy-related 5.

The distributions of Parkin and ATG5 levels are presented in *Figure 3*. Both displayed non-normal distributions, with the median Parkin level measured at 203 (118–401) pg/mL and the median ATG5 level at 83 (52–101) ng/mL. Plasma levels of Parkin were higher in the very late-onset recurrence group than in the non-very late-onset recurrence group [225.0 (136.5–491.5) pg/mL vs. 136.0 (92.3–237.0) pg/mL, *P* = 0.038]. In contrast, ATG5 levels did not differ significantly between the two groups [82.8 (47.9–101.5) ng/mL vs. 78.4 (61.1–106.4) ng/mL, *P* = 0.70]. The comparison of baseline characteristics by median Parkin level is presented in *Table 4*. Serum creatinine level was significantly higher in the below-median group compared to the above-median group (*P* = 0.013).

**Table 4:** Comparison of baseline characteristics by median Parkin level

Variable	Above the median	Below the median	*P*-value
Demographics			
Age (years)	71 [63–77]	69 [57–76]	0.206
Female, *n* (%)	40 (40)	35 (34)	0.374
Body mass index (kg/m^2^)	23.2 ± 4.1	23.6 ± 4.2	0.568
Persistent atrial fibrillation, *n* (%)	40 (40)	44 (43)	0.694
CHA2DS2-VASc	3 [1–4]	2 [1–4]	0.302
Lifestyle			
Daily drinking	26 (26)	29 (28)	0.730
Daily smoking	18 (18)	14 (14)	0.389
Comorbidities			
History of heart failure admission, *n* (%)	35 (35)	30 (29)	0.370
Hypertension, *n* (%)	54 (54)	59 (57)	0.638
Diabetes mellitus, *n* (%)	20 (20)	11 (11)	0.065
Ischaemic stroke, *n* (%)	8 (8)	10 (10)	0.669
Vascular disease, *n* (%)	2 (2)	2 (2)	0.976
Aetiology of heart disease			
Ischaemia, *n* (%)	9 (9)	5 (5)	0.244
Dilated cardiomyopathy, *n* (%)	10 (10)	13 (13)	0.556
Valvular heart disease, *n* (%)	11 (11)	4 (4)	0.053
Cardiac implantable electronic devices, *n* (%)	3 (3)	3 (3)	0.971
Pacemaker, *n* (%)	2 (2)	2 (2)	0.976
Cardiac resynchronization therapy, *n* (%)	1 (1)	1 (1)	0.309
Medications			
ACEi or ARB, *n* (%)	46 (46)	50 (49)	0.717
ARNI, *n* (%)	6 (6)	4 (4)	0.486
β-Blockers, *n* (%)	67 (67)	63 (61)	0.386
Mineralocorticoid receptor antagonists, *n* (%)	16 (16)	19 (18)	0.645
Sodium-glucose cotransporter-2 inhibitors, *n* (%)	10 (10)	16 (16)	0.238
Echocardiographic parameters			
Left atrial diameter (mm)	41 [35–46]	42 [37–47]	0.397
LV ejection fraction (%)	64 [57–69]	61 [53–70]	0.303
Laboratory data			
Serum creatinine (mg/dL)	0.82 [0.70–0.98]	0.90 [0.78–1.05]	0.013
N-terminal pro-brain natriuretic peptide (pg/mL)	446 [163–983]	425 [98–1079]	0.876
Sources of ablation energy			0.059
Radiofrequency, *n* (%)	48 (48)	36 (35)	
Cryoballoon, *n* (%)	52 (52)	67 (65)	
Strategies of the procedures			
Pulmonary veins isolation only, *n* (%)	62 (62)	75 (73)	0.100
Additional lines			
Posterior wall isolation, *n* (%)	20 (20)	15 (15)	0.305
Roof block line utilized by cryoballoon, *n* (%)	12 (12)	19 (18)	0.202
Cavotricuspid isthmus block line, *n* (%)	6 (6)	13 (13)	0.105
Superior vena cava isolation, *n* (%)	1 (1)	4 (4)	0.185
Medications at discharge			
Any anti-arrhythmic drugs, *n* (%)	17 (17)	13 (13)	0.380
Sodium channel blockers, *n* (%)	5 (5)	2 (2)	0.233
Bepridil, *n* (%)	4 (4)	3 (3)	0.671
Amiodarone, *n* (%)	8 (8)	8 (8)	0.951

The abbreviations are the same as those in *Table 1*.

**Figure 4:**
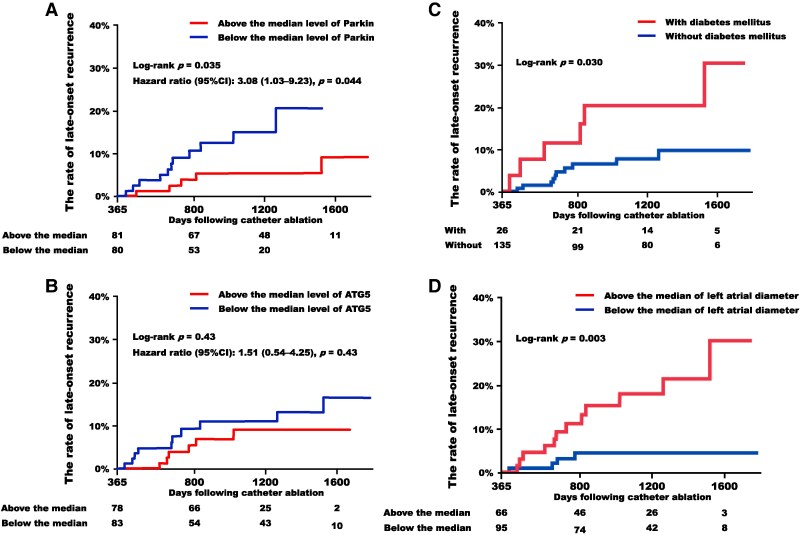
Kaplan–Meier curves for very late-onset recurrence. (*A*) Comparison of Parkin levels below and above the median. (*B*) Comparison of ATG5 levels below and above the median. (*C*) Comparison between patients with and without diabetes mellitus. (*D*) Comparison of left atrial diameter below and above the median. CI, confidence interval; other abbreviation is the same as *Figure 3*.

Kaplan–Meier curves for the very late-onset recurrence, stratified by each biomarker with its median value used as the cut-off to compare groups above and below the median, are presented in *Figure 4*. The group with Parkin levels below the median, indicative of suppressed mitophagy, demonstrated a statistically significantly higher rate of very late-onset recurrence compared to the group with levels above the median (*Figure 4A*). In contrast, no such difference was observed with ATG5 levels (*Figure 4B*).

The results of the Cox proportional hazards regression analysis for each baseline characteristic are presented in *Table 5*. Among these, diabetes mellitus and left atrial diameter were found to be associated with very late-onset recurrence [hazard ratio (95% confidence interval): 2.93 (1.06–8.12), *P* = 0.038, and 1.11 (1.04–1.19), *P* = 0.002, respectively]. Multivariable analysis, incorporating Parkin levels below the median, diabetes mellitus, and left atrial diameter, revealed a statistically significant association for Parkin levels in predicting very late-onset recurrence [hazard ratio (95% confidence interval): 3.82 (1.20–12.13), *P* = 0.023]. Very late-onset recurrence-free survival curves stratified by diabetes mellitus and left atrial diameter are presented in *Figure 4C* and *D*, respectively.

**Table 5:** Predictive factors of late-onset atrial fibrillation recurrence

	Univariable			Multivariable		
	HR	95% CI	*P*-value	HR	95% CI	*P*-value
Age (1-year increase)	1.02	0.97–1.07	0.48			
Female	0.81	0.28–2.33	0.69			
BMI (1 kg/m^2^ increase)	0.96	0.82–1.09	0.53			
Persistent atrial fibrillation	0.66	0.21–2.05	0.47			
Pulmonary veins isolation only	0.65	0.23–1.80	0.41			
Anti-arrhythmic drugs at discharge	0.96	0.22–4.24	0.96			
CHA2DS2-VASc (1 point increase)	1.31	0.99–1.71	0.05			
Lifestyle						
Daily drinking	0.94	0.30–2.93	0.92			
Daily smoking	0.73	0.16–3.21	0.67			
Comorbidities						
History of heart failure admission, *n* (%)	2.06	0.76–5.54	0.15			
Hypertension, *n* (%)	2.58	0.83–8.02	0.10			
Diabetes mellitus, *n* (%)	2.93	1.06–8.12	0.038	3.51	1.23–9.99	0.019
Ischaemic stroke, *n* (%)	3.26	0.93–11.45	0.07			
Medications						
ACEi, ARB, or ARNI	1.83	0.66–5.04	0.24			
β-Blockers	0.62	0.23–1.65	0.34			
Mineralocorticoid receptor antagonists	2.18	0.69–6.86	0.18			
Sodium-glucose cotransporter-2 inhibitors	0.59	0.08–4.50	0.61			
Echocardiographic parameters						
Left atrial diameter (1 mm increase)	1.11	1.04–1.19	0.002	1.11	1.03–1.19	0.005
LV ejection fraction (1% increase)	0.99	0.95–1.03	0.54			
Laboratory data						
Creatinine (1 mg/dL increase)	1.35	0.23–4.29	0.70			
N-terminal pro-brain natriuretic peptide (1 increase)	1.00	1.00–1.00	0.57			
Parkin levels below the median	3.08	1.03–9.23	0.044	3.82	1.20–12.13	0.023

Other abbreviations are the same as those in *Table 1*. Cox proportional hazards regression analysis was performed to identify factors associated with the primary endpoint.

CI, confidence interval; HR, hazard ratio.

### Parkin and the secondary endpoint

**Figure 5:**
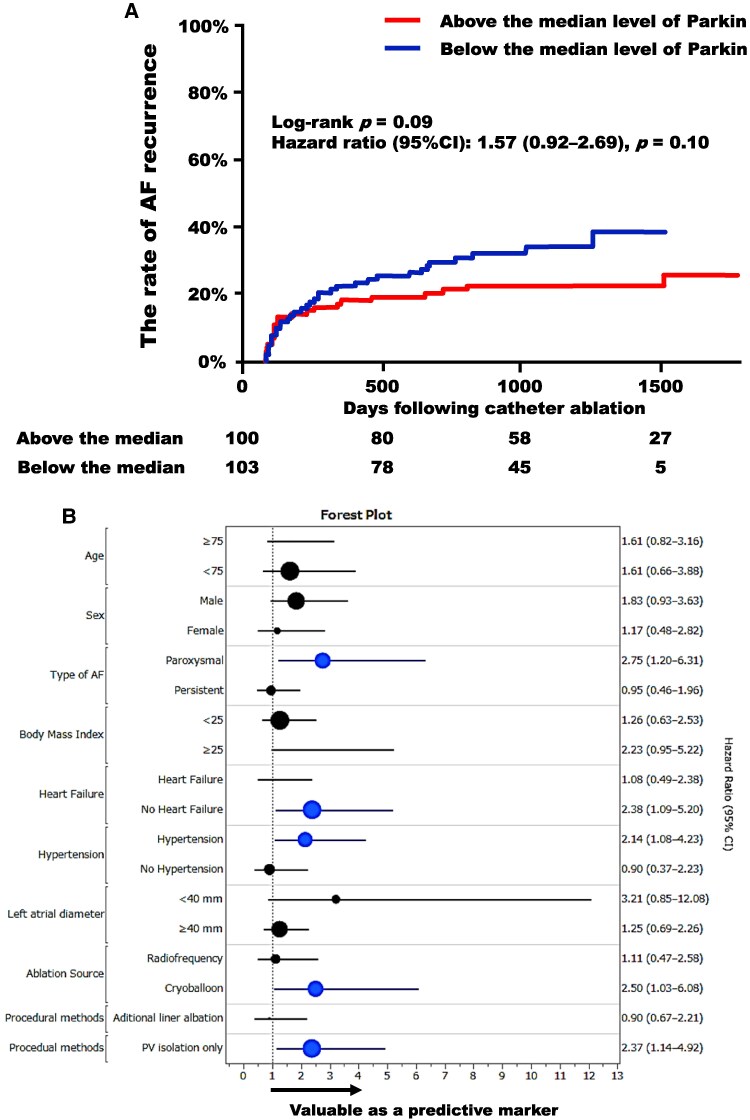
Kaplan–Meier curves for atrial fibrillation recurrence following catheter ablation. (*A*) Comparison of Parkin levels below and above the median. (*B*) Forest plot of atrial fibrillation recurrence by subgroups. The size of the points reflects the number of patients. Blue plots indicate groups for which *P*-values were below 0.05. AF, atrial fibrillation; PV, pulmonary vein; other abbreviation is the same as *Figure 4*.

Kaplan–Meier curves for the AF recurrence following catheter ablation are presented in *Figure 5A*. Similarly, the group with Parkin levels below the median tended to have a higher rate of recurrence compared to the group with levels above the median; however, this difference did not reach statistical significance (log-rank *P* = 0.09).

The association between Parkin levels and AF recurrence following catheter ablation was analysed through subgroup analysis, considering background factors commonly associated with AF recurrence, and a forest plot was generated (*Figure 5B*). Parkin levels were found to have predictive value in subjects with paroxysmal AF (not persistent type), those without a history of heart failure admission, those with coexisting hypertension, those treated with cryoballoon instead of radiofrequency, and those undergoing pulmonary vein isolation only.

## Discussion

This study represents the first clinical investigation to elucidate the impact of mitochondrial autophagy on the pathophysiology of AF recurrence. In the initial feasibility study, a reduction in γ-glutamyl dipeptides was observed in the very late-onset recurrence group. In a subsequent study involving all enrolled subjects, a decrease in serum Parkin levels, a key mediator of defective mitochondrial clearance, was found to predict very late-onset recurrence following catheter ablation. In contrast, serum ATG5 levels, a marker of bulk autophagy, showed no association with very late-onset recurrence.

### Pathophysiological significance of metabolome analysis

Several clinical factors have been identified as contributors to AF recurrence following the procedure, including gender differences, left atrial enlargement, persistent type, inflammation, hypertension, and metabolic syndrome.^14–17^ To counter these recurrence factors, several ablation techniques, including linear ablation, posterior wall isolation, low voltage area ablation, and non-PV foci induction, have been investigated. However, none of these approaches have resulted in consistently satisfactory outcomes.^18–22^ Therefore, it can be stated that the time has come for us to focus not only on improving procedural techniques but also on elucidating the molecular biological mechanisms underlying AF recurrence, constructing optimal risk stratification, and identifying novel therapeutic targets.

However, the key biological players remain unclear. Chronic inflammation, including that associated with metabolic syndrome, has been implicated in the occurrence of AF.^23^ Although high-sensitivity C-reactive protein, white blood cell count, and fibrinogen are commonly utilized as representative markers of chronic inflammation, their utility in predicting recurrence after AF ablation remains a matter of ongoing debate.^24^ Therefore, a non-targeted analysis of metabolic alterations, including those associated with chronic inflammatory reactions, may provide a more comprehensive insight into its pathophysiology.^25^

Metabolomic analysis in this study identified numerous metabolites, among which a decrease in γ-glutamyl dipeptides was associated with very late-onset recurrence. While previous studies proposed a hypothesis suggesting an increase in γ-glutamyl dipeptides during starvation and certain disease states, our findings demonstrate a decrease in these dipeptides in the recurrence group, reflecting the opposite condition.^26,27^ We hypothesized that insufficient synthesis of γ-glutamyl dipeptides may contribute to the very late-phase recurrence of AF. Although the detailed biosynthetic pathway of γ-glutamyl peptides has not been fully elucidated, it has been reported that some, particularly γ-glutamylcysteine, are synthesized in the mitochondria and possess antioxidant properties.^28^ Moreover, extracellular γ-glutamyl dipeptides are known to modulate intracellular calcium concentrations through the calcium-sensing receptor.^29^ In the experimental study, pathogenic hypertrophy of cardiomyocytes was attenuated by inhibition of the calcium-sensing receptor, a key mediator of extracellular calcium levels, due to the suppression of autophagy activity resulting from altered calcium handling.^30^ A reduction of extracellular γ-glutamyl dipeptides, as observed in our findings, may contribute to the inhibition of autophagy activity.

### Interpretation of the impact of autophagy on AF recurrence

In our study, a low serum level of Parkin, a representative marker of mitophagy, was associated with very late-onset recurrence, as well as left atrial enlargement and the coexistence of diabetes mellitus. Although it is assumed that the serum concentration of Parkin reflects systemic changes rather than heart-specific fluctuations in this study, the observed association with AF recurrence constitutes an important step in elucidating its pathophysiology. The fact that a significant difference was observed only with Parkin, rather than with bulk autophagy activity, was an important finding that highlights the crucial role of mitochondrial quality control in the recurrence of AF.

Previous studies have demonstrated that abnormalities in mitochondrial-related calcium handling are involved in the progression of AF.^31,32^ Our findings revealed a reduction in serum γ-glutamyl dipeptides, which are synthesized in the mitochondria, in the group with very late-onset recurrence.^28^ These findings may be interpreted as the result of the accumulation of dysfunctional mitochondria due to reduced mitophagy, leading to a reduction in γ-glutamyl dipeptides and impaired calcium handling, which ultimately contributes to AF recurrence.

In contrast to Parkin, the ATG5 level did not demonstrate any predictive value for very late-onset recurrence in this study. ATG5 is widely recognized as a representative marker of non-selective autophagy.^33^ These findings suggest that the regulation of mitochondria-selective autophagy may play a role in the mechanisms underlying very late-onset recurrence.

### Clinical implications of serum Parkin level measurement

Although the serum level of Parkin was a strong predictor of very late-onset recurrence, its impact was not evident within approximately 1 year following catheter ablation, as demonstrated by the secondary endpoint results. While many previous studies on AF ablation have assessed outcomes within 1 year of the procedure, our findings highlight the need for longer observational periods to fully elucidate the ‘true’ outcomes following catheter ablation.^34,35^ In standard clinical practice, there are cases where anticoagulation therapy is discontinued if no AF recurrence is observed during the 1-year follow-up period. However, the findings of this study suggest that, in cases with low Parkin levels prior to the procedure, extended observation or continued anticoagulation therapy may be warranted. Additionally, metformin and rapamycin, which have been reported as promoters of mitophagy, may prove effective in suppressing recurrence in patients with low Parkin levels; however, further investigation is required.^36,37^

The results of the sub-analysis were also intriguing, as Parkin levels were found to be useful in predicting recurrence in the group that underwent pulmonary vein isolation alone; however, subjects who underwent additional linear ablation procedures did not demonstrate this trend (*Figure 5B*). These findings may suggest that low Parkin levels reflect non-pulmonary vein foci inducing AF, which may indicate the progression of left atrial remodelling.

### Study limitations

This study has several limitations. First, it was conducted at a single centre, and procedural methods varied. However, these variations are likely negligible in the analysis of AF recurrence, as there is no conclusive evidence supporting the efficacy of ablation beyond pulmonary vein isolation. Second, due to limitations in research funding, the number of samples available for metabolomic analysis was restricted. Third, very late-onset recurrence may have been under-detected in some subjects due to the challenges of identifying AF recurrence without continuous cardiac rhythm monitoring. Fourth, given the observational design of the study, it remains unclear whether variations in Parkin levels were a consequence of AF outcomes or a causal factor and whether targeting Parkin could play a role in preventing recurrence. Furthermore, given the various reports on biomarkers, it remains unclear whether Parkin can serve as an independent predictor, as not all biomarkers were measured in this study. Lastly, the mechanisms underlying the association between Parkin and very late-onset recurrence, as opposed to recurrence within 1-year post-ablation, remain uncertain. The findings of this study suggest that the mechanisms underlying recurrence within the first year following ablation may differ from those associated with recurrence occurring thereafter. As γ-glutamyl dipeptides were found to be reduced in the very late-onset AF recurrence group, further studies are needed to determine whether glutamate supplementation could improve mitochondrial autophagy function.

## Conclusions

Metabolome analysis suggested the association between autophagy and recurrence following catheter ablation for AF. Serum levels of Parkin, which is a representative biomarker of mitophagy, predicted the very late-onset recurrence. Further research is warranted to elucidate the mechanisms underlying AF development mediated by mitochondrial autophagy.

## Data Availability

The data sets used for this study are not publicly available but are available from the corresponding author on adequate request.
